# Dynamics of Strong Coupling Between Free Charge Carriers in Organometal Halide Perovskites and Aluminum Plasmonic States

**DOI:** 10.3389/fchem.2021.818459

**Published:** 2022-01-14

**Authors:** Yang Luo, Hai Wang, Le-Yi Zhao, Yong-Lai Zhang

**Affiliations:** State Key Laboratory of Integrated Optoelectronics, College of Electronic Science and Engineering, Jilin University, Changchun, China

**Keywords:** strong coupling, rabi splitting, perovskite, aluminum plasmonic states, transient absorption spectra, free charge carriers

## Abstract

We have investigated a strong coupled system composed of a MAPbI_x_Cl_3-x_ perovskite film and aluminum conical nanopits array. The hybrid states formed by surface plasmons and free carriers, rather than the traditional excitons, is observed in both steady-state reflection measurements and transient absorption spectra. In particular, under near upper band resonant excitation, the bleaching signal from the band edge of uncoupled perovskite was completely separated into two distinctive bleaching signals of the hybrid system, which is clear evidence for the formation of strong coupling states between the free carrier–plasmon state. Besides this, a Rabi splitting up to 260 meV is achieved. The appearance of the lower bands can compensate for the poor absorption of the perovskite in the NIR region. Finally, we found that the lifetime of the free carrier–SP hybrid states is slightly shorter than that of uncoupled perovskite film, which can be caused by the ultrafast damping of the SPs modes. These peculiar features on the strong coupled hybrid states based on free charge carriers can open new perspectives for novel plasmonic perovskite solar cells.

## Introduction

Organometal halide perovskites are emerging as a class of attractive materials for solution-processed optoelectronic devices with outstanding performance.(Jeon et al., 2015; Yang et al., 2015; Long et al., 2020; Chen et al., 2021; Privitera et al., 2021). Perovskites possess the same crystal structure of calcium titanate ABX_3_ (D. Weber and Naturforsch, 1978), where A and B are typical organic cations (methylammonium or formamidinium) and metal cations (Pb^2+^ or Sn^2+^) jointly bound to X, halide anions (Cl^−^, Br^−^ or I^−^). Through compositional control of such components and ratios, various perovskite materials can be realized (Zhao and Zhu, 2016; Correa-Baena et al., 2017). Owing to the specific crystal structure, perovskites show great advantages, such as high absorption coefficient, high charge-carrier mobility, low trap density, and long charge diffusion length (Stranks et al., 2013; Xing et al., 2013). Simultaneously, through improving the architecture of the photovoltaic devices, a power conversion efficiency (PCE) of up to 25.2% is achieved (Yoo et al., 2021). Methylammonium (MA) lead trihalide perovskites with the chemical composition CH_3_NH_3_PbX_n_Y_3–n_ is most widely and maturely researched. Their fundamental parameters, such as photoexcited charge-carrier dynamics and carrier diffusion length, have been deeply explored. In transient absorption measurements, a long charge diffusion length >1 μm is revealed in CH_3_NH_3_PbCl_x_I_3–x_ perovskite films (Stranks et al., 2013). Furthermore, the nature of photogenerated species has also been expounded carefully. It is confirmed that, upon photoexcitation, excitons within the perovskite can be spontaneously dissociated into free electrons and holes, namely, conventional excitons can be displaced by free charge carriers, which are identified as dominated photogenerated species in perovskite film (Manser and Kamat, 2014). These characteristics bring many distinct advantages, making the OLHP available to high-performance solar cells, light-emitting diodes, and photodetectors.

Moreover, great progress has also been made in using surface plasmons (SPs) to further enhance the performance of plasmonic devices based on OLHP (Fang et al., 2010; Bi et al., 2013; Ren et al., 2020; Juan et al., 2021; Krisnanda et al., 2021). Affected by the SPs, the optoelectronic characteristics of the perovskite film can be significantly tuned, such as enhancement of absorption, reduction of exciton binding energy, increase of charge mobility, and so on (Saliba et al., 2015; Chan et al., 2017; Ding et al., 2018; Ma et al., 2019; Mohamed Saheed et al., 2019; Krisnanda et al., 2021). However, most of the works still survive in the weak coupling regime in which the wave function of the perovskite material is unperturbed. In contrast, only a few reports have emerged on the strong coupling between SPs and OLHP. In a strong coupling regime, the excitation energy can be rapidly exchanged between SPs modes and materials with the formation of new hybrid states separated energetically by a Rabi splitting (Qian et al., 2021; Su et al., 2021; Zhao et al., 2021). In this regime, the new states are coherently super-positioned with intriguing phenomena, such as Bose–Einstein condensation (Plumhof et al., 2014) and thresholdless lasing (Ding and Ning, 2012; Wang et al., 2021). Among the variety of research, many types of excitonic materials, for instance, J-aggregates (Hao et al., 2011; Wang et al., 2016), dye molecules (Wang et al., 2017), quantum dots (Wang et al., 2016), and two-dimensional material (Liu et al., 2016; Shan et al., 2019) are realized in a strong coupling regime with SPs. In contrast, so far little is known about the coherent hybrid system generated by the interaction of free charge carriers and SPs. Particularly, there is still limited understanding on the kinetics of hybrid SP–free charge carrier states. It is also worth emphasizing again that, on account of the small exciton binding energies, free charge carriers characterized by slow recombination and relatively high mobility can be efficiently generated in OLHP (Manser and Kamat, 2014). Thus, there is a strong incentive to harness such materials for studying strong coupling between SPs and free charge carriers.

In this work, we demonstrate that strong SP–free charge carrier coupling can be achieved with CH_3_NH_3_PbCl_x_I_3–x_ perovskite film and SPs supported by Al conical nanopits arrays. Furthermore, under upper band resonant excitation, direct evidence of the formation of the hybrid free charge carrier–SP states is clearly shown in the transient absorption (TA) spectra. The result also proves that the band filling of the upper states follows the Burstein–Moss band filling model. Finally, we measured a shorter lifetime of the hybrid SP–free charge carrier states, which was accelerated by the ultrafast damping of the SPs character.

## Experimental Section

**FIGURE 1:**
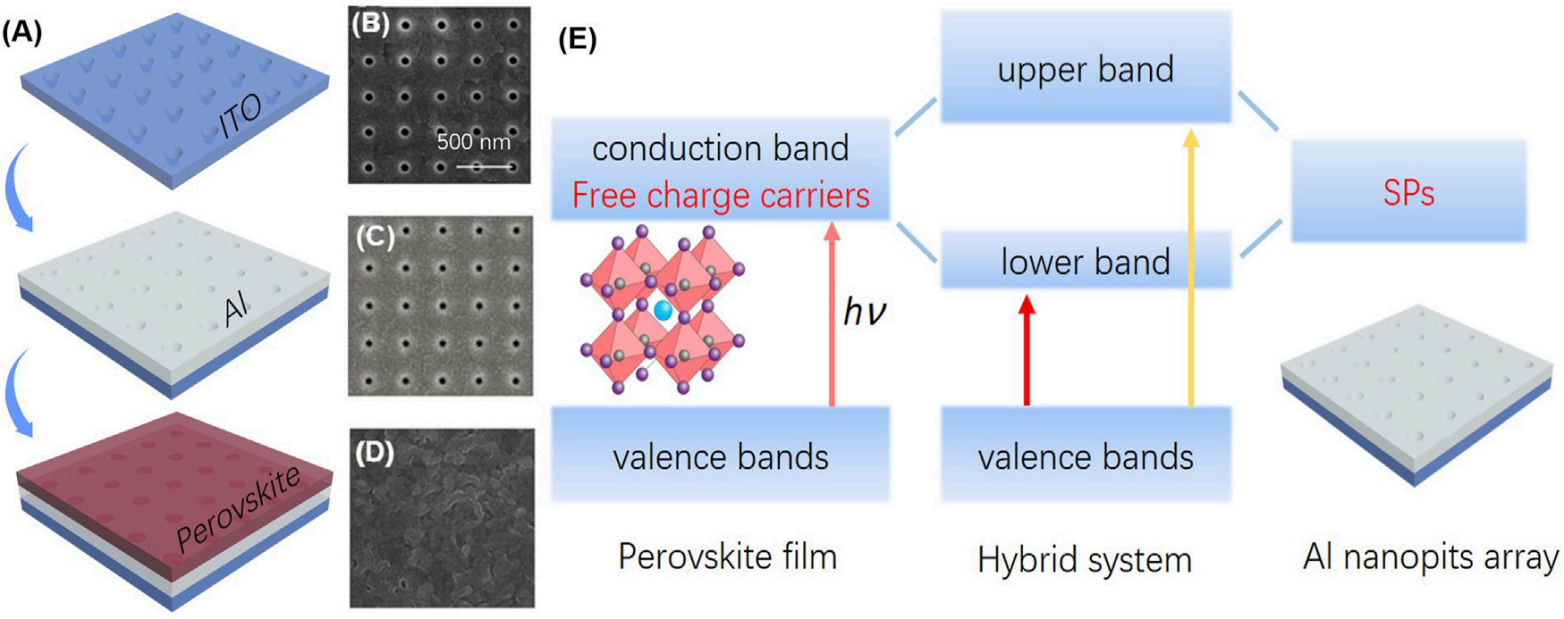
**(A)** Steps of fabrication of strong free charge carrier–SP coupling system, **(B, C)** SEM image of the conical nanopits on glass/ITO and Al layer, **(D)** SEM image of perovskite layer, **(E)** the mode diagram of the hybrid free charge carrier–SP system; the inset shows the crystal structure of CH_3_NH_3_PbCl_x_I_3-x_ perovskite.

The fabrication processes of the strong SP–free charge carrier coupled system is described in Figure 1A. First, the cleaned glasses/ITO was milled by means of a focused ion beam to define the shape of a square-like array of conical pits. Afterward, an electron beam evaporator was used for the 200-nm-thick aluminum (Al) film deposition on the patterned glasses/ITO substrates. Then, conical pit arrays with a period from 300 to 450 nm can be replicated on top of the Al film with a total area of 50 × 50 μm^2^. Al was selected not only simply for its cost-effectiveness and abundance, but also for the wide SP spectral range (Li et al., 2016). Here, the processed Al nanopits with uniform size (as shown in Figure 1C) show a plasmon resonance that matches with the broad absorbance spectrum of CH_3_NH_3_PbCl_x_I_3-x_ film. To obtain a strong coupled hybrid system between SPs and free charge carriers, the perovskite films were directly formed on Al conical nanopits. The perovskite precursor solution was prepared by dissolving 139.0 mg of PbI_2_ and 238.5 mg of CH_3_NH_3_I in 2 ml N,N-dimethylformamide (DMF). Then, the samples were obtained by spin-coating these precursor solutions at 3000 rpm for 30 s and annealed at 80°C for 100 min in a nitrogen-filled glove box. Figure 1D provides SEM images of the morphology of the synthesized CH_3_NH_3_PbI_x_Cl_3-x_ film with grain sizes ranging from 200 to 250 nm CH_3_NH_3_PbCl_x_I_3-x_ and CH_3_NH_3_PbI_3_ have similar optical properties, such as absorption spectrum, bandgaps, and Fermi levels. However, it is demonstrated that the Cl-doped perovskite film has a long charge carrier diffusion length, always leading to a higher efficiency solar cell (Xing et al., 2013). Here CH_3_NH_3_PbCl_x_I_3-x_ is selected rather than CH_3_NH_3_PbI_3_.

### Steady-State Measurements

**FIGURE 2:**
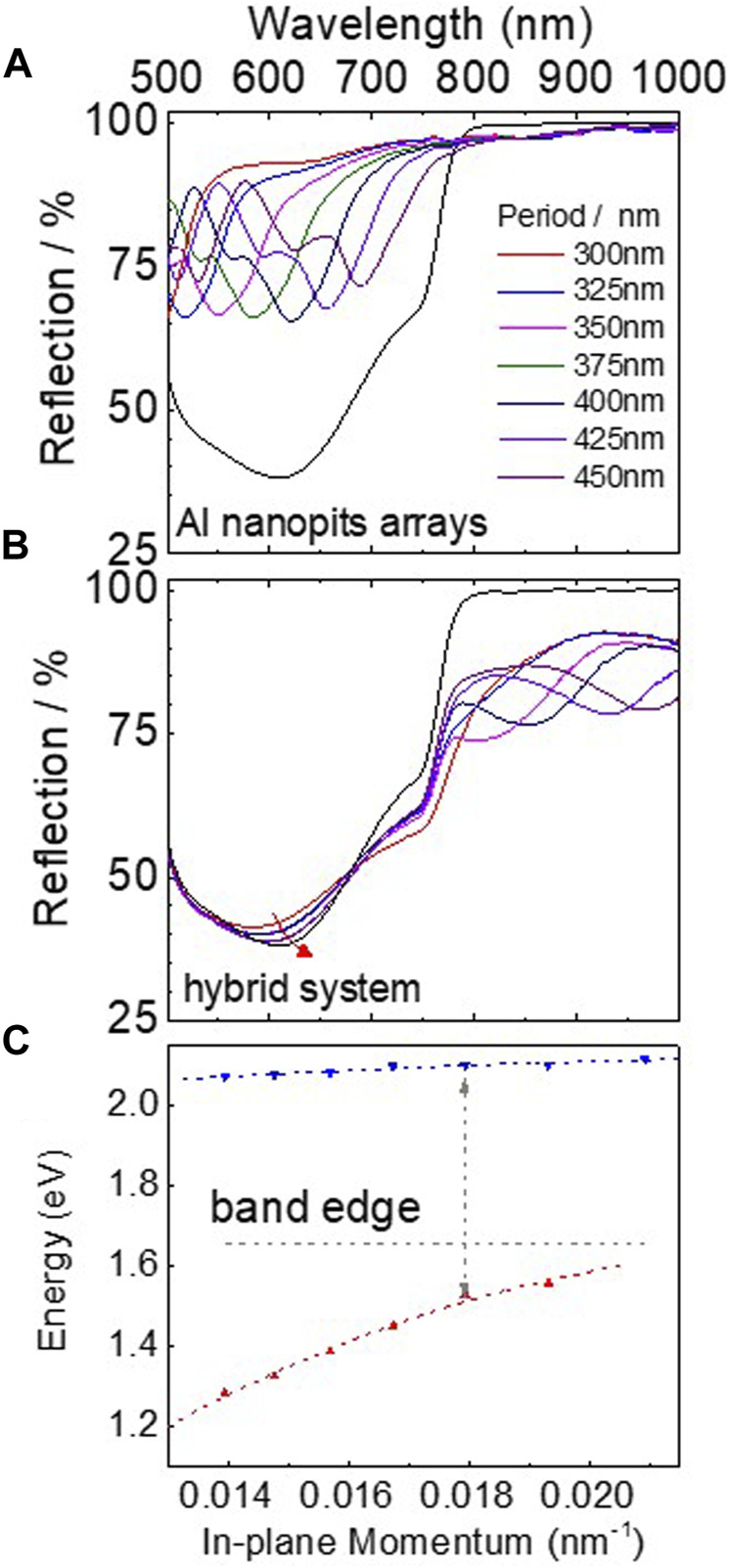
**(A)** Reflection spectra of Al conical nanopit arrays covered by PMMA film. The lattice period is tuned from 300 to 450 nm. The black line shows the reflection spectrum of the CH_3_NH_3_PbCl_x_I_3-x_ perovskite film on flat Al film. **(B)** Reflection spectra of the perovskite layer on flat Al film and conical nanopit arrays. The lattice period is tuned from 300 to 450 nm. **(C)** Dispersion curve of hybrid free change carrier–SP strong coupled system. The horizontal dashed line shows the band edge of the CH_3_NH_3_PbCl_x_I_3-x_ perovskite film.

Figure 2A shows the reflection spectra of Al nanopit arrays with various periods. The reflection peaks display a pronounced red shift as the period of the nanopit arrays increases, showing the SP resonance region from 490 to 750 nm. In the meantime, for each nanopit array, a shoulder peak appeared on the higher energy side of the main SPs peak. The offset of the two peaks can be attributed to the difference of the refractive index of the media above and below the Al film. The reflection spectrum of the CH_3_NH_3_PbCl_x_I_3-x_ perovskite film deposited on flat Al film is also shown in Figure 2A as a black line. As can be seen, the perovskite film has a strong optical absorption with a band edge at 1.65 eV. Observably, all the SP bands overlap within the reflection spectrum of CH_3_NH_3_PbCl_x_I_3-x_ film. Thus, when perovskite film is formed on Al nanopit arrays, the hybrid system is prepared.

A key point to underlined here is the demonstration that free charge carriers generated in perovskite film and SPs in the hybrid system can enter the strong coupled regime. Because the initial study on strong coupling was focused on a series of J-aggregate molecules with unusually strong transition dipoles, which was considered as the only possible candidate for strong coupling. However, moving forward, much larger Rabi splitting has also been achieved between SPs and molecules with broad absorption spectra, such as photochromic molecules and semiconductor polymer (Schwartz et al., 2011; Orgiu et al., 2015). Actually, several studies demonstrate that the vacuum Rabi splitting depends on the energy-integrated absorption of the material, namely, a larger vacuum Rabi splitting can be achieved by using material with higher absorption cross-sections (Gambino et al., 2014). Therefore, in the following part, a clear indication of strong coupling between SPs and free charge carriers in CH_3_NH_3_PbCl_x_I_3-x_ perovskite film is provided.

Figure 2B shows a series of reflection spectra of the hybrid system with different Al nanopit periods. Compared with the perovskite film on flat Al film, the reflection spectra of the hybrid systems are significantly changed. In particular, at the wavelength longer than the band edge of the perovskite, extra reflection peaks corresponding to the lower bands are shown, which can lead to overcoming the poor absorption of the CH_3_NH_3_PbCl_x_I_3-x_ film in the NIR region. On the other side, due to the strong absorption in the visible range of the perovskite film, the reflection spectra of the hybrid system at the higher energy side only changed slightly. However, it can be seen that the peak position related to the upper bands also has a red shift with the lattice period increasing as the red arrow shown in Figure 2B. Furthermore, the dispersion diagram of the two bands is shown in Figure 2C. As expected, anticrossing behavior as remarkable experimental evidence of strong coupling is clearly observed (Dintinger et al., 2005).

However, because most light is absorbed by the perovskite layer when the wavelength (λ) of the light is shorter than the band edge of CH_3_NH_3_PbCl_x_I_3-x_ (λ < 760 nm), the formation of the upper bands in the hybrid systems cannot be clearly reflected from reflection spectra. The evidence of the formation of the new hybrid SP–free charge carrier states is not comprehensive. The Rabi splitting energy cannot be revealed just from steady-state measurements. Therefore, transient absorption spectroscopy was performed to gain a further insight into the formation and dynamics of the hybrid states.

### TA experiments

**FIGURE 3:**
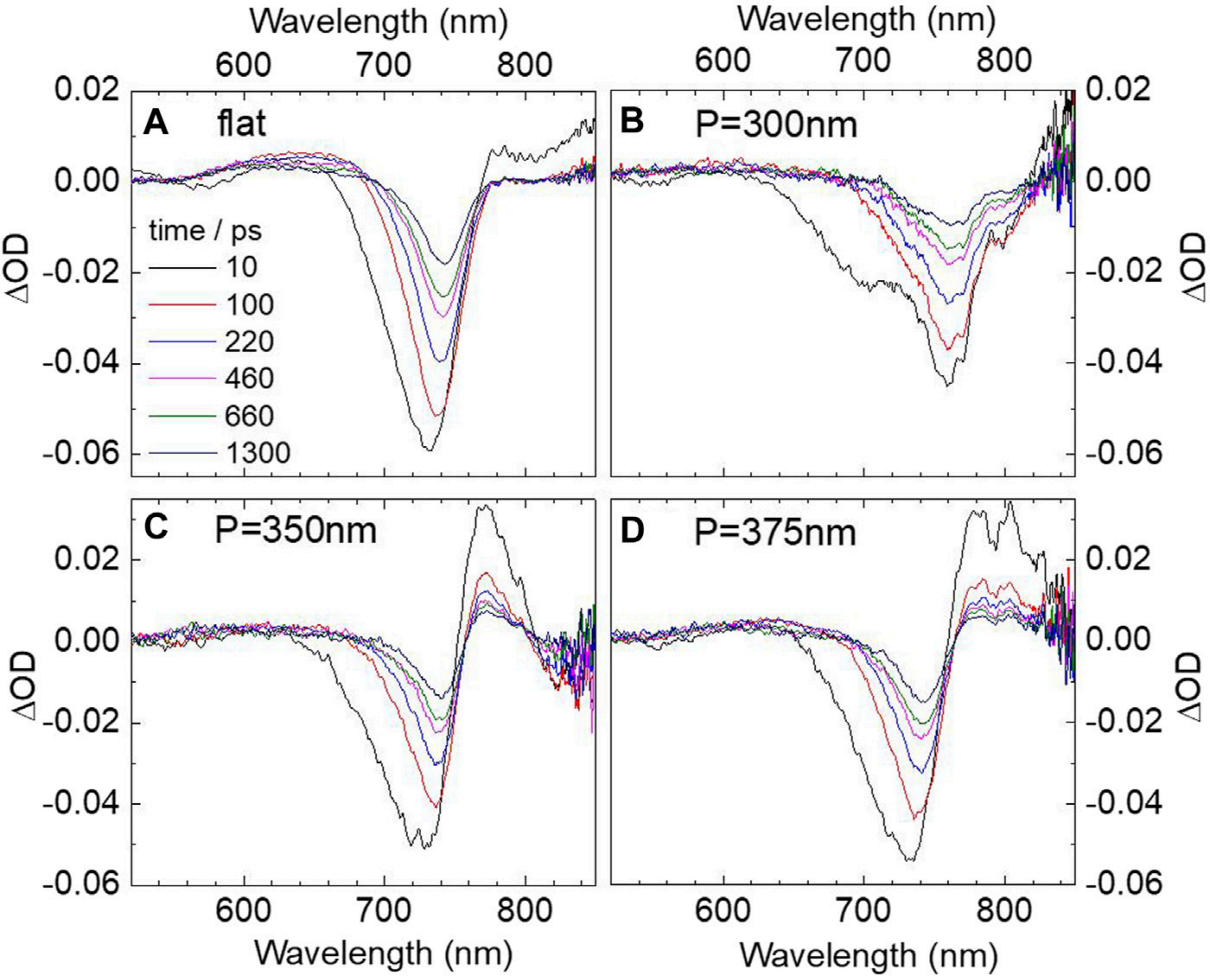
Transient absorption spectra of CH_3_NH_3_PbCl_x_I_3-x_ perovskite film on flat **(A)** Al film and **(B–D)** different Al nanopit arrays with periods of 300, 350, and 375 nm under 400 nm excitation. The spectra are recorded at 10, 100, 220, 460, 660, and 1300 ps.

TA spectroscopy, also named the pump-probe technique (Gao et al., 2010; Wang et al., 2013), is an effective approach to the study of charge carrier transfer and recombination dynamics of perovskite films (Zhang et al., 2015; Zhang et al., 2016). Femtosecond reflective TA spectra was carried out by a 100 fs laser pump-probe system. A Ti:sapphire amplifier system was used to provide an 800-nm wavelength laser with 100 fs pulse width and 500 Hz repetition rate. The amplified output was split into two parts. The stronger part was sent to a β-barium borate (BBO) crystal or TOPAS system to generate the pump pulse at 400 or 560 nm while the weaker part was focused on a sapphire crystal to create a continuous white light as a probe beam. The relative delay between the pump and probe beam was controlled by an optical delay line (Newport DL325). Then, the two beams were focused on the sample by a microscope objective. The probe beam reflected from the sample was collected by an optical spectrometer (Avantes AvaSpec -ULS2048CL-EVO) while the reflected pump beam was filtered. Here, we focused on the TA spectroscopy findings on the nature of the hybrid SP–free charge carrier states. To begin with, the first part of the TA measurements were performed under nonresonant conditions by the 400 nm laser pulse. Figure 3A shows the TA spectra of CH_3_NH_3_PbCl_x_I_3-x_ film on flat Al film. The figure consists of a negative band at 730 nm corresponding to band edge ground state bleaching, and on both sides of the bleaching, there are two positive signals due to the photoinduced absorption of hot electrons and holes (Herz, 2016). In addition, it is worth noting that the high-energy tail of the band edge bleaching spectrum is broadened and shows a blue shift in the first 10 ps, which can be explained by a dynamic Burstein−Moss shift (Manser and Kamat, 2014). Namely, after laser excitation, the conduction and valence band edges are filled with thermalized carriers; further considering the Pauli exclusion principle, the occupation of band-edge states is pushed to higher energies, leading to a dynamic Burstein–Moss band filling model.

Figures 3B–D shows the corresponding spectra of CH_3_NH_3_PbCl_x_I_3-x_ film on Al nanopit arrays with different periods of 300, 350, and 375 nm. The spectra are a little changed compared with the reference sample on flat Al film. First, for the period equal to 300 nm, the spectra show a negative signal at 760 nm and a shoulder peak at 700 nm, which are possibly corresponded to the hybrid bands. However, the separation between the two new peaks is much smaller than that measured from steady-state reflection spectra, so the strong coupling regime still cannot be clearly identified. For the period equal to 350 and 375 nm, Figures 3C,D shows a bleaching band around 730 nm, which is still dominated by the ground-state bleaching from uncoupled CH_3_NH_3_PbCl_x_I_3-x_ film. On the red side of the bleaching band, a positive signal is observed corresponding to the thermal effect of the SPs (Hao et al., 2011) while the bleaching signals from the upper bands are also almost undetectable. In fact, similar to the previous work under nonresonant excitation (Hao et al., 2011; Wang et al., 2016), the intrinsic photophysics of the strong coupled hybrid system cannot be observed. Under such conduction, the initial relaxation process is dominated by a state-filling process and SP thermal effect as the positive signal reported in Supplementary Figure S1.

**FIGURE 4:**
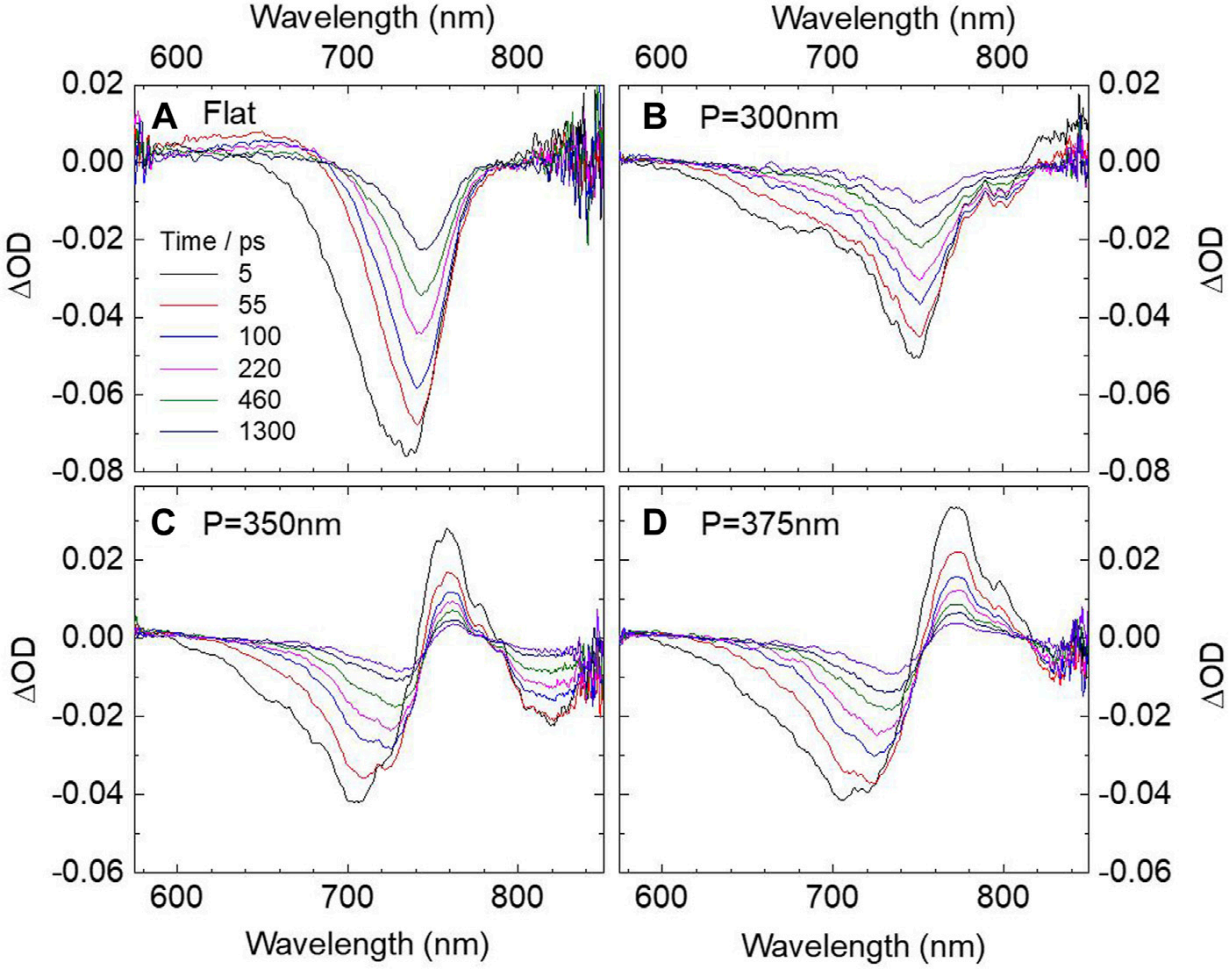
Transient absorption spectra of CH_3_NH_3_PbCl_x_I_3-x_ perovskite film on flat **(A)** Al film and **(B–D)** different Al nanopit arrays with periods of 300, 350, and 375 nm under 560 nm excitation. The spectra are recorded at 5, 55, 100, 220, 460, and 1300 ps.

**FIGURE 5:**
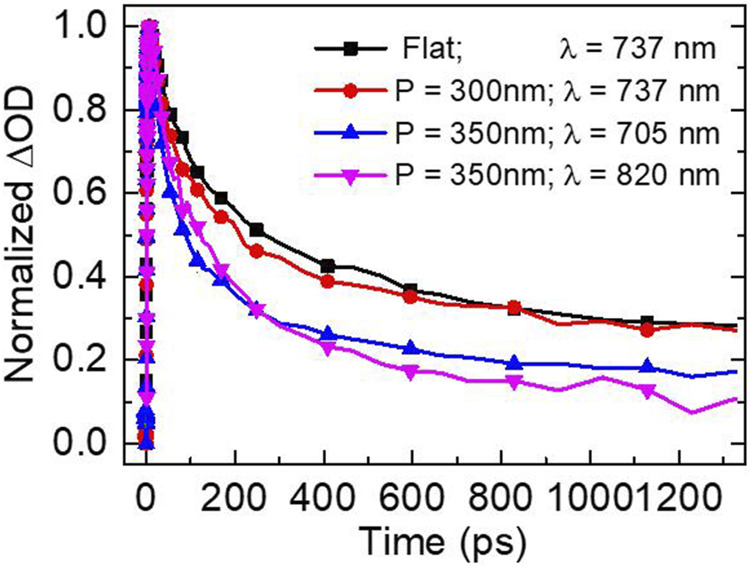
Normalized bleaching dynamics under 560 nm resonant excitation of CH_3_NH_3_PbCl_x_I_3-x_ perovskite film on a flat Al film (measured at 737 nm) and on Al nanopit arrays with periods of 350 nm (measured at the hybrid bands of 705 and 820 nm) and 300 nm (measured at the upper band of 737 nm).

To explore the nature photophysics of the hybrid SP–free charge carrier states more deeply, TA measurements were further carried out under near upper state resonant excitation by a pulsed laser at 560 nm. Figure 4A illustrates the TA spectra of CH_3_NH_3_PbCl_x_I_3-x_ film on flat Al film. As a reference, the plot is similar to the spectra obtained under nonresonant excitation at 400 nm. A bleaching signal around 730 nm, which also conforms to the Burstein–Moss band filling model, shows a carrier accumulation–induced blue shift (Manser and Kamat, 2014) while the small positive signals below 680 nm are assigned to photoinduced absorption. The spectra for CH_3_NH_3_PbCl_x_I_3-x_ film on different periodic nanopit arrays are reported in Figures 4B–D, which more clearly suggest the formation of strong coupled hybrid SP–free charge carrier states. First, when the nanopit period equals 300 nm, the spectra show a main bleaching peak at 750 nm, corresponding to the lower hybrid band. On the contrary, the amplitude of the upper hybrid band is much smaller, showing a shoulder peak around 670 nm. Under this condition, the splitting was not obvious, which suggests the coupling strength is low. This can be further proved by the dynamic behaver of the hybrid states (see Figure 5), which is still similar to the bleaching recovery of pure perovskite film.

More importantly, when the nanopit period equals 350 nm, the SP resonance of Al nanopits can resonate with the free charge carriers in perovskite film. With respect to the reference sample, the TA spectra of the hybrid system show completely different properties. The band edge transition bleaching is completely separated into two bleaching signals at around 700 and 820 nm, which can be attributed to the upper and lower hybrid SP–free charge carrier states with observation of Rabi splitting energy up to 260 meV. Such behavior provides more objective, precise evidence than that observed from steady-state reflection measurements. With the period further increased to 375 nm, as shown in Figure 4D, the bleaching peak of the upper state is a little red shifted while the bleaching band of the lower state also experiences a red shift almost extending out of the detection window. By comprehensive analysis of the three different period hybrid systems, the simultaneous shifting of both upper and lower hybrid states is consistent with the characteristic strong coupling: anticrossing behavior, which further remarkably underlines the formation of the coherent hybrid SP–free charge carrier states. Another important observation that needs to be stressed here is that, under upper band resonant excitation, the lower band bleaching signal is narrowing with time. Such distinguishable narrowing of the lower band can also be described by the Burstein–Moss effect. On the other hand, the shape of the lower hybrid states does not change with the time. We, therefore, attribute that free carriers are accumulated at the lower bands of the hybrid states after 560 nm laser excitation. The understanding presented here provides new mechanisms of strong coupling between SPs and free charge carriers, which serve to shed light on designing novel ultrafast plasmonic devices.

Finally, the kinetic process of the hybrid SP–free charge carrier states is analyzed. In Figure 5, we plot the amplitude-normalized dynamics of the lower and upper hybrid bands of the coupled sample with a nanopit period of 350 nm compared with the dynamics of the band edge bleaching of perovskite film on a flat Al film. As shown, under upper band resonant excitation, the population dynamics of the hybrid states are shorter than the bleaching recovery of free charge carriers in pure perovskite film. This result show interesting differences from the lifetime measured from hybrid states composed of traditional excitons and SPs. Previous experiments confirm that the longer lifetimes of the hybrid states is intrinsic and can be even longer than the lifetime of bare excitons, which is accorded with a non-Markovian regime (Canaguier-Durand et al., 2015). However, here we should emphasize that, again, the excited-state population in CH_3_NH_3_PbCl_x_I_3-x_ film is dominated by free charge carriers. The ultrafast damping plasmon character (Pelton et al., 2008; Hartland, 2011) reduces the lifetime of the coherent hybrid SP–free charge carrier states. Namely, the relaxation of such new hybrid states can be expedited by SPs, leading to a shorter lifetime.

## Conclusion

In summary, we construct a hybrid structure comprising an Al subwavelength nanopit array and perovskite film. In steady-state measurements, the reflection spectra profile of the hybrid systems characterized by two distinct bands, whose dispersions show the typical signature of strong coupling. These results experimentally demonstrate that strong coupling can be achieved with SPs and free charge carriers generated in CH_3_NH_3_PbCl_x_I_3-x_ film. Moreover, the nature photophysics of the new hybrid SP–free charge carrier states was studied by TA spectroscopy. Under nonresonant excitation by a 400-nm laser, the initial relaxation process is dominated by a state-filling effect, which still cannot provide the intrinsic dynamics of the hybrid state. Furthermore, under near upper band resonant excitation, the TA spectra remarkably displayed the formation of the hybrid SP–free charge carrier state, in which the upper bands can be well ascribed to the dynamic Burstein–Moss effect. We have also found that the lifetime of the hybrid SP–free charge carrier states is shorter than the bleaching recovery of pure CH_3_NH_3_PbCl_x_I_3-x_ film, which is different from that observed in a strong coupling system with traditional excitons conformed to a non-Markovian regime. As a possible explanation, we assume that the ultrafast damping of the SP modes accelerates the relaxation of the hybrid SP–free charge carrier states. To conclude, these new insights on strong coupling with hybrid organic-inorganic perovskites provide a new framework to develop low-cost photovoltaic and light-emitting devices and nanolasers (Sanvitto and Kena Cohen, 2016). New developments on nanoplasmonic devices working in a strong coupling regime with free charge carriers are surely on the horizon.

## Data Availability

The original contributions presented in the study are included in the article/Supplementary Material, further inquiries can be directed to the corresponding author.
