# Supplementary material for: Dynamics of Strong Coupling Between Free Charge Carriers in Organometal Halide Perovskites and Aluminum Plasmonic States
Source: Front Chem. 2022 Jan 14;9:818459. doi: 10.3389/fchem.2021.818459 (PMC8795516; doi:10.3389/fchem.2021.818459)
Supplement: Supplementary file 1 [file DataSheet1.docx]

Supplementary Material


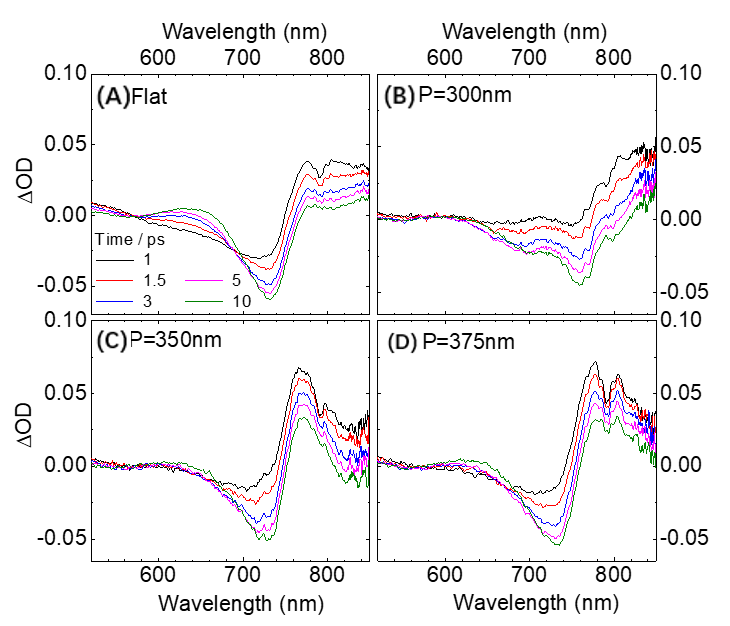


**Supplementary Figure S1.**. Transient absorption spectra of CH_3_NH_3_PbCl_x_I_3-x_ perovskite film on flat (A) Al film and (B-D) different Al nanopits arrays with periods of 300, 350, and 375 nm under 400 nm excitation. The spectra are recorded at 1, 1.5, 3, 5, and 10 ps.
